# Brain microvascular pericytes are immunoactive in culture: cytokine, chemokine, nitric oxide, and LRP-1 expression in response to lipopolysaccharide

**DOI:** 10.1186/1742-2094-8-139

**Published:** 2011-10-13

**Authors:** Andrej Kovac, Michelle A Erickson, William A Banks

**Affiliations:** 1 Geriatrics Research Education and Clinical Center, Veterans Affairs Puget Sound Health Care System, Seattle, Washington, USA; 2 Division of Gerontology and Geriatric Medicine, Department of Internal Medicine, University of Washington, Seattle, Washington, USA; 3 Department of Pharmacological and Physiological Sciences, Saint Louis University School of Medicine, St. Louis, MO USA; 4 Institute of Neuroimmunology, Slovak Academy of Sciences, Bratislava, Slovakia

**Keywords:** mouse brain pericytes, LPS, neurovascular unit, cytokines, chemokines, LRP-1, Alzheimers disease, nitric oxide

## Abstract

**Background:**

Brain microvascular pericytes are important constituents of the neurovascular unit. These cells are physically the closest cells to the microvascular endothelial cells in brain capillaries. They significantly contribute to the induction and maintenance of the barrier functions of the blood-brain barrier. However, very little is known about their immune activities or their roles in neuroinflammation. Here, we focused on the immunological profile of brain pericytes in culture in the quiescent and immune-challenged state by studying their production of immune mediators such as nitric oxide (NO), cytokines, and chemokines. We also examined the effects of immune challenge on pericyte expression of low density lipoprotein receptor-related protein-1 (LRP-1), a protein involved in the processing of amyloid precursor protein and the brain-to-blood efflux of amyloid-β peptide.

**Methods:**

Supernatants were collected from primary cultures of mouse brain pericytes. Release of nitric oxide (NO) was measured by the Griess reaction and the level of S-nitrosylation of pericyte proteins measured with a modified "biotin-switch" method. Specific mitogen-activated protein kinase (MAPK) pathway inhibitors were used to determine involvement of these pathways on NO production. Cytokines and chemokines were analyzed by multianalyte technology. The expression of both subunits of LRP-1 was analyzed by western blot.

**Results:**

Lipopolysaccharide (LPS) induced release of NO by pericytes in a dose-dependent manner that was mediated through MAPK pathways. Nitrative stress resulted in S-nitrosylation of cellular proteins. Eighteen of twenty-three cytokines measured were released constitutively by pericytes or with stimulation by LPS, including interleukin (IL)-12, IL-13, IL-9, IL-10, granulocyte-colony stimulating factor, granulocyte macrophage-colony stimulating factor, eotaxin, chemokine (C-C motif) ligand (CCL)-3, and CCL-4. Pericyte expressions of both subunits of LRP-1 were upregulated by LPS.

**Conclusions:**

Our results show that cultured mouse brain microvascular pericytes secrete cytokines, chemokines, and nitric oxide and respond to the innate immune system stimulator LPS. These immune properties of pericytes are likely important in their communication within the neurovascular unit and provide a mechanism by which they participate in neuroinflammatory processes in brain infections and neurodegenerative diseases.

## Background

The blood-brain barrier (BBB) is a selective barrier that is created by the endothelial cells in cerebral microvessels. Endothelial cells and supporting cells such as astrocytes, pericytes, neurons, and perivascular microglia are organized together to form the "neurovascular unit" which is essential for induction, function, and support of the BBB [1]. In contrast to the considerable knowledge characterizing the crosstalk among brain endothelial cells, astrocytes, and microglia within the neurovascular unit during inflammation, very little is known about the role played by the brain microvascular pericyte.

Among the cells of the neurovascular unit, brain microvascular pericytes are physically the cells closest to brain endothelial cells, wrapping around them, joined to them by gap junctions, and interfacing with them by peg-and-socket structures [2,3]. These cells are also essential for the induction of the barrier properties of the BBB and attrition of pericytes during the neovascularization process [4] or aging [5] can lead to increased vascular permeability. Furthermore, it has been described that pericytes regulates BBB-specific gene expression in endothelial cells and induces polarization of astrocyte end-feets [6].

The exact contribution of pericytes to regulation of brain blood capillary flow is still not adequately examined. Early ultrastructural studies showed that cerebellar pericytes contains microfilaments similar to actin- and myosin-containing muscle fibers [7,8]. Furthermore, it has been described that at least some subpopulations of brain pericytes express contractile proteins such as α-smooth muscle actin and non-muscle myosin [9,10]. More recently, using the acute brain tissue preparation, Peppiatt et al., showed dilatation of cerebellar pericytes as an response to glutamate stimulation [11]. Studies on cultured pericytes support contractile role of these cells however the expression of contractile proteins such as α-smooth muscle actin seems to be changed after cultivation [12].

Several in-vitro studies exist that demonstrated that pericytes are multipotent cells. Pericytes isolated from adult brains can differentiate into cells of neural lineage [13]. Cultured brain pericytes express macrophage markers ED-2 and CD11b and to exhibit phagocytic activity, thus expressing immune cell properties [14].

During pathological conditions such as sepsis, pericytes detach from the basal lamina which leads to increased cerebrovascular permeability. Activation of pericytes through TLR-4 has been suggested to be responsible for this process [15].

Here, we focused on the immunological profile of cultured mouse brain pericytes in the quiescent and immune-challenged state. We studied production of immune mediators such as nitric oxide (NO), cytokines, and chemokines. We also examined the effects of immune activation on pericyte expression of low density lipoprotein receptor-related protein-1 (LRP-1), an immune-modulated processor of amyloid precursor protein and a brain-to-blood efflux pump for amyloid beta peptide.

## Methods

### Mouse brain pericytes culture

Primary mouse brain microvascular pericytes were prepared according to Nakagawa et al [16]. Briefly, cultures of mouse cerebrovascular pericytes were obtained by a prolonged, 2-week culture of isolated brain microvessel fragments, containing pericytes and endothelial cells. Pericyte survival and proliferation was favored by selective culture conditions using uncoated dishes and DMEM F12 supplemented with 20% fetal calf serum (Sigma, USA), L-glutamine (2 mM, GIBCO, USA) and gentamicin (Sigma, USA). Culture medium was changed twice a week.

### Cell stimulation

Mouse brain microvascular pericyte cultures (p2-p8) were stimulated with lipopolysaccharide from *Salmonella typhimurium *(L6511; Sigma, USA) for 4, 8, and 24 hours. For MAPK pathways study, SB203580 (p38 MAPK inhibitor, Tocris, USA), PD98059 (MAPKK/MEK inhibitor, Tocris, USA), UO126 (MEK-1/MEK-2 inhibitor, Tocris, USA), SP600126 (c-Jun N-Terminal kinase inhibitor, Sigma, USA) and PTDC (NF-κB inhibitor, Sigma, USA) were added to the pericytes cultivated in 96 well plates 1 h before cell stimulation with LPS.

### Nitrite assay and detection of S-nitrosylated proteins

Nitrite, a downstream product of nitric oxide (NO), was measured by the Griess reaction in culture supernatants as an indicator of NO production. Briefly, 50 ul of cell culture medium was incubated with 100 ul of Griess reagent A (1% sulfanilamide, 5% phosphoric acid; Sigma, USA) for 5 min, followed by addition of 100 ul of Griess reagent B (0.1% N-(1-naphtyl) ethylenediamine; Sigma, USA) for 5 min. The absorbance was determined at 540 nm using a microplate reader.

Assessment of S-nitrosylation was done by a modification of the "biotin-switch" method. Cells were washed in PBS and lysed in lysis buffer contain NEM (*N*-ethylmaleimide) to block free thiol groups. *S*-nitrosothiols were then reduced, biotinylated and visualized after SDS-PAGE/western blot using a streptavidin-based detection system (Cayman Chemical Company, USA). Membranes were digitalized with a LAS4000 CCD imaging system (GE Healthcare, USA) and analyzed by ImageQuant TL software.

### Measurement of cytokines and chemokines

Concentrations of cytokines and chemokines secreted into the culture media were measured by a commercial magnetic bead based Multiplex ELISA kit (Bioplex, Biorad, USA) according to the manufacturer's protocol.

### Immunocytochemistry

Pericytes grown on glass cover slips (12 mm diameter) were washed in PBS and fixed with 4% PFA for 10 min at 4°C. Cells were permeabilized with 0.2% TRITON-X100, blocked with 5% BSA, and then incubated with anti-α smooth muscle actin antibody (Abcam, USA), anti-CD13 antibody (Abcam, USA), *Griffonia simplicifolia *lectin-FITC (Sigma, USA), anti-factor VIII antibody (Sigma, USA) and anti-GFAP antibody (Abcam, USA) followed by incubation with corresponding ALEXA Fluor-488 or Alexa Fluor-546 conjugated secondary antibody (Invitrogen-Molecular Probes, USA). Finally, slides were mounted in fluorescence mounting media and photographed with a Nikon ECLIPSE E800 fluorescence microscope.

### Western blotting

For LRP-1, pericyte extracts were run on a 3-8% Tris-acetate gel (non-reducing conditions), transferred onto nitrocellulose membranes (Invitrogen, USA), and probed first with a LRP-1 primary antibody that recognizes the large subunit (Sigma, USA) and then with a LRP-1 primary antibody that recognizes the small subunit (Epitomics, 2703-1). SYPRO Ruby (Invitrogen, USA) staining of membranes was used to verify uniformity of protein loading [17]. Incubation with primary antibodies was followed by horseradish peroxidase-conjugated secondary antibody (Santa Cruz, USA). As positive and negative controls, respectively, MEF-1 (SV40 transformed mouse embryo fibroblasts, ATCC, USA) and PEA-13 (mouse embryo fibroblasts, ATCC, USA) cell lysates were loaded onto the gel. The enhanced chemiluminescence western blot was digitalized with a LAS4000 CCD imaging system (GE Healthcare, USA) and analyzed by ImageQuant TL software.

### Data analysis

Values are presented as means ± SEM. More than two means were compared by one-way ANOVA followed by Tukey's multiple comparison test (Prism 5.0 software, GraphPad, inc, San Diego, CA). Differences at P < 0.05 were accepted as statistically significant.

## Results

### Characterization of purity of primary mouse brain pericyte cultures

**Figure 1:**
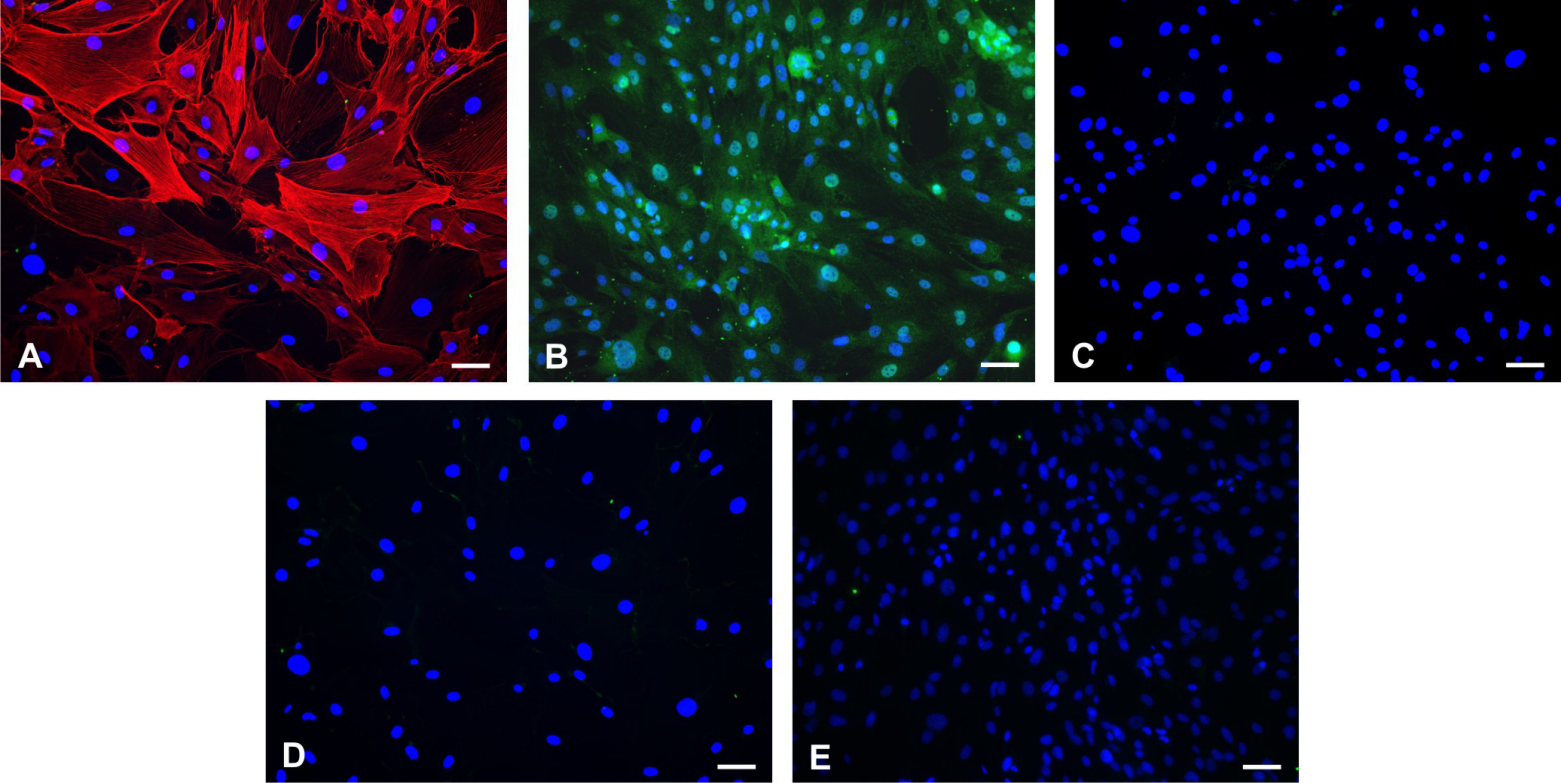
**Determination of the purity of the pericyte culture**. A primary culture of pericytes isolated from mouse brain microvessels was labeled with anti-α smooth muscle actin antibody (pericyte marker; red) (Panel A), anti-CD13 antibody (pericyte marker; green) (Panel B), anti-GFAP antibody (astrocytes marker; green) (Panel C), *Griffonia simplicifolia *lectin (microglial marker; green) (Panel D) or anti-factor VIII antibody (endothelial cell marker; green) (Panel E) and counterstained with nuclear stain DAPI (blue). Visual observation of immunostained cells in pericyte cultures demonstrates that they primarily consist of a α-smooth muscle actin/CD13 positive pericytes. No contamination with microglia, astrocytes or endothelial cells was detected. Scale bar: 40 μm.

Purity of isolated primary mouse brain pericytes was analyzed by immunocytochemical staining of cultures. We evaluated the presence of contaminating astrocytes, microglia and endothelial cells. More than 95% of cells in cultures was positive for the pericyte markers α-smooth muscle actin [14,18] (Figure 1A) and CD13 (aminopeptidase N) [19-22] (Figure 1B). Results demonstrated that there was no contamination of our primary pericyte cultures either with astrocytes (Figure 1C), microglia (Figure 1D) or endothelial cells (Figure 1E).

### LPS induces nitric oxide production via MAPK pathways in mouse brain pericytes

**Figure 2:**
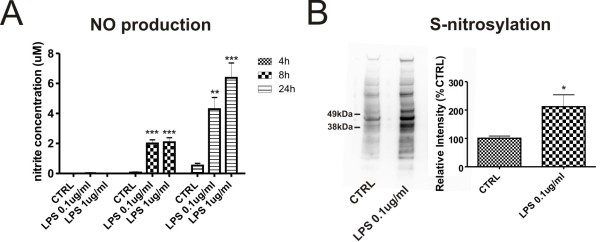
**Release of nitric oxide and nitrosative stress in primary brain pericytes after LPS stimulation**. Brain pericytes were stimulated for 4, 8, and 24 h with LPS (0.1 and 1 ug/ml), media collected, and analyzed for NO production by the Griess reaction. LPS (0.1 ug/ml and 1 μg/ml) induced a significant NO release from cells after 8 and 24 hours (A). Nitrative stress was accompanied by massive S-nitrosylation of cellular proteins (B). Values of nitrite accumulation from treated cells represent the mean ± SEM of two independent experiments conducted in tetraplicates. *P < 0.05, **P < 0.01, ***P < 0.001 vs. untreated cells.

Activation of immune cells is accompanied by production of different immune mediators. Thus, we studied the effect of LPS on production of nitric oxide (NO) and various cytokines and chemokines by cultured primary brain pericytes. Pericytes were treated for 4, 8 and 24 h with different concentrations of the LPS and nitrite (a downstream product of NO) concentration in cell culture media was measured. LPS at concentrations of 0.1 and 1 μg/ml after 8 and 24 h significantly induced NO release (for example, 24 h results: controls: 0.5 ± 0.15 uM at 24 h; 0.1 ug/ml LPS: 4.3 ± 0.77 uM; 1 ug/ml LPS: 6.4 ± 0.98 uM; n = 8/group). There was no change in NO production at 4 h. (Figure 2A) Production of reactive nitrogen species led to increased S-nitrosylation of pericyte proteins (2.4× in 0.1 ug/ml LPS vs CTRL, n = 3) (Figure 2B).

**Figure 3:**
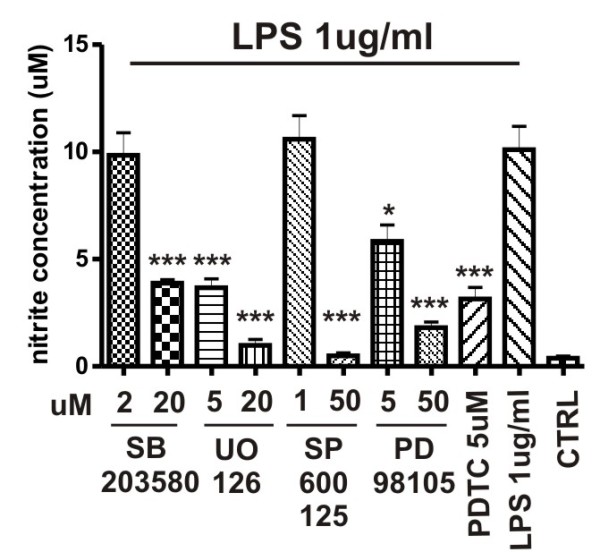
**Involvement of MAPK pathways in nitric oxide production by pericytes after LPS stimulation**. Brain pericytes were stimulated for 4, 8, and 24 h with LPS (0.1 and 1 ug/ml). MAPK pathway inhibitors were added to the culture medium 1 h before LPS treatment. Media was collected and analyzed for NO production by Griess reaction. Addition of MAPK pathways inhibitors significantly reduced NO production by LPS treated pericytes. Values represent the mean ± SEM of two independent experiments conducted in tetraplicates. *P < 0.05, ***P < 0.001 vs. untreated cells.

To identify the signal transduction pathway responsible for production of reactive nitrogen species, we tested several MAPK inhibitors and the NF-κB inhibitor PDTC for their ability to reduce NO production by pericytes. Pre-incubation of cells with SB203580 (at 20 uM; p38 MAPK inhibitor), PD98059 (at 5 and 50 uM; MAPKK/MEK inhibitor), UO126 (at 5 and 20 uM; MEK-1/MEK-2 inhibitor), SP600126 (at 50 uM; c-Jun N-Terminal kinase inhibitor) and PTDC (at 5 uM) significantly inhibited production of NO by cultured brain pericytes (Figure 3). These results indicated involvement of the MAPK signaling pathway in LPS-induced NO production.

### LPS stimulates cytokine and chemokine release by primary mouse brain pericytes

**Figure 4:**
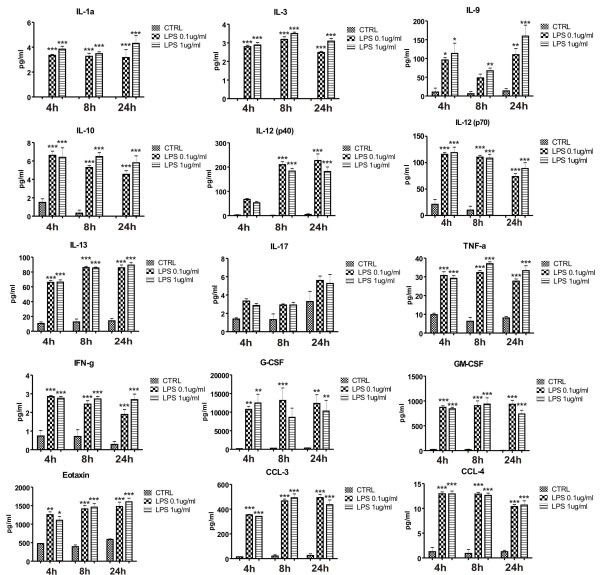
**Release of cytokines and chemokines from primary brain pericytes constitutively and after LPS stimulation**. Brain pericytes were stimulated for 4, 8, and 24 h with LPS (0.1 and 1 ug/ml). Media was collected and cytokine and chemokine concentrations were determined via commercial magnetic bead immunoassay. Addition of LPS at 0.1 ug/ml concentration induced significant changes in production of several pro-inflammatory cytokines and chemokines from brain pericytes. Values of cytokine production represent the mean ± SEM of two independent experiments conducted in triplicates *P < 0.05, **P < 0.01, ***P < 0.001 vs. untreated cells.

Pericytes spontaneously released several interleukins (IL), including IL-9, IL-10, IL-12(p70), IL-13, and IL-17. Levels of IL-1 alpha, IL-3, and IL-12(p40) were not detectable. Other cytokines and chemokines that were detected were tumor necrosis factor-alpha, interferon-gamma, granulocyte-colony stimulating factor, granulocyte macrophage-colony stimulating factor, eotaxin, CCL-3 and CCL-4. To further characterize pericyte immune capacity, we determined the effect of LPS on the release of cytokines and chemokines. The results (Figure 4) showed that stimulation of primary mouse brain pericyte cultures with 0.1 and 1 ug/ml LPS resulted in significant release of pro-inflammatory cytokines such as IL-1α, TNF-α, IL-3, IL-9 and IL-13 (4 h, 8 h and 24 h) and anti-inflammatory cytokines such as IL-10 (4 h, 8 h, 24 h). Additionally, LPS-stimulated pericytes significantly increased their secretion of IL12 heterodimer (p70) and of its p40 subunit. Moreover, activated pericytes produced more chemokines such as G-CSF, eotaxin, CCL-3, CCL-4 (4 h, 8 h and 24 h) and MCP-1, KC, CCL-5 (4 h, 8 h, 24 h; data not shown) in comparison to unstimulated control cells. Of the detected cytokines, only the increase in IL-17 was not significant. There was no detectable constitutive or LPS-induced production of IL-1b, IL-2, IL-4 and IL-5 by brain pericytes.

### LPS induces up-regulation of LRP-1 expression in brain pericytes

**Figure 5:**
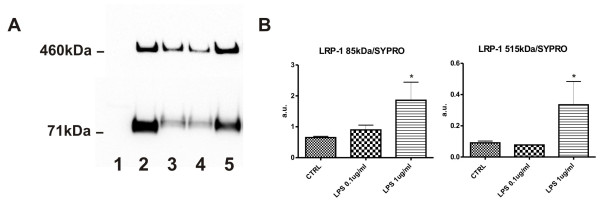
**LPS induce up-regulation of LRP-1 expression in brain pericytes**. Primary brain pericytes were stimulated for 24 h with LPS (0.1 and 1 ug/ml). After 24 h, expression of both LRP-1 subunits was analyzed by western blot as described in the *Material and methods*. LPS at 1 ug/ml concentration induced significant increases in expression of the large (515 kDa) and small (85 kDa) subunits of LRP-1. A representative western blot (A) and density quantification (B) based on ratios between the antibody signal (LRP-1 85 or 515 kDa) and total protein loading per lane (SYPRO) is shown. Lane designation: 1-PEA13 (LRP-1 knockout as negative control), 2-MEF1 (LRP-1 wild type as positive control), 3-CTRL, 4-LPS 0.1 ug/ml, 5-LPS 1 ug/ml. Values represent the mean ± SEM of two independent experiments * P < 0.05 vs. untreated cells, n = 5.

Neuroinflammation plays an important role in neurodegeneration. Here, we analyzed the effect of LPS on expression of LRP-1 in pericytes. Stimulation of cells with LPS (1 ug/ml) for 24 hours significantly increased expression of both subunits of LRP-1 protein (Figure 5A representative WB and quantification Figure 5B). The MEF1 (LRP-1 wild type) and PEA13 (LRP-1 knockout) cells were used as positive and negative controls respectively for LRP-1 antibodies.

## Discussion

In this work, we focused on the characterization of the immunological properties of mouse brain pericytes under inflammatory conditions induced by LPS. We have used primary mouse brain pericytes as a model cell culture for our studies. These cells were isolated by modifications of the method for isolation of microcapillaries from mouse brains. However, such isolation procedures potentially can lead to cultures that are contaminated with adjacent cell types such as astrocytes, endothelial cells, and juxtavascular microglia; furthermore, the presence of these contaminating cells can lead to erroneous results [23,24]. Staining with markers for microglia, astrocytes and endothelial cells that are not expressed by pericytes [18], showed that our cultures were free of these cell types.

Nitric oxide (NO) is a signaling molecule and immune mediator that is released from glial and endothelial cells with activation. Microglia and astrocytes are common sources of NO in the brain during CNS inflammatory processes [25]. Production of large amounts of NO by iNOS-2 can lead to generalized nitrosative stress in cells and to posttranslational modification of protein residues by S-nitrosylation. S-nitrosylation mediates many of the biological effects of NO. This posttranslational modification causes specific physiological or pathophysiological activities by modifying protein thiols [26]. S-nitrosylated of peptides or proteins are involved in many human diseases such as type II diabetes, Alzheimer's disease, and Parkinson's disease [27]. Our results demonstrated that LPS strongly induces production of nitric oxide and nitrosative stress in brain pericytes. Furthermore, we found increased S-nitrosylation of pericyte proteins. It will be important to further analyze and study those pericyte proteins which are affected by increased S-nitrosylation of their thiol residues.

Mitogen-activated protein kinase (MAPK) signal transduction pathways belong to the most prevalent mechanisms of eukaryotic cells that respond to extracellular stimuli [28]. We used several MAPK pathway inhibitors to analyze the involvement of these pathways in the release of nitric oxide by brain pericytes in response to LPS. Our results clearly showed that production of NO was blocked by pre-incubation of pericytes with these drugs. These results agree with those obtained from lung microvascular pericytes [29] and indicate that similar mechanisms are involved in activation of brain microvascular pericytes by LPS.

Another interesting finding of our study is related to the production of important signaling molecules, cytokines and chemokines by pericytes. Of 23 cytokines and chemokines that we studied, 18 were secreted by brain pericytes constitutively or in response to LPS stimulation. LPS is derived from the bacterial coat of gram negative bacteria and is a strong stimulant of the innate immune system. Among the several cytokines and chemokines whose production was increased by LPS, IL-12, IL-13, and IL-9 are of particular interest with regard to pericyte communication within the neurovascular unit. IL-12 plays a critical role in the early inflammatory response to infection. An increased production of IL-12 is involved in the pathogenesis of a number of autoimmune inflammatory diseases (multiple sclerosis, arthritis, insulin dependent diabetes) [30-32]. IL-12 consists of two subunits (p40 and p35) which are linked together by a disulfide bond to give heterodimeric p70 molecule [33]. We showed that brain pericytes release substantial amounts of both the heterodimeric p70 molecule and p40 subunits after LPS stimulation. Release of the p40 subunit was higher than release of the heterodimeric p70 molecule itself. Interestingly, the p40 subunit of IL12 can link together and this homodimeric form has been shown to increase expression of leukocyte chemoattractant factor (IL-16) in microglia [34].

IL-9 is another pleiotropic cytokine whose production was markedly increased after LPS stimulation of brain pericytes. IL-9 is mainly produced by T lymphocytes and mediates allergic inflammation in tissues such as the lung and intestine [35]. In the CNS, the IL-9 receptor complex is present on astrocytes and IL-9 stimulated astrocytes express CCL-20 chemokine which promotes infiltration of Th17 cells into the CNS [36].

IL-13 is known as an anti-inflammatory cytokine that is produced by microglia but not astrocytes or neurons after direct injection of LPS into the cortex. Neurons are required for IL-13 production by microglia and production of IL-13 by microglia leads to the death of activated microglia and enhancement of neuronal survival [37]. In our study, IL-13 production by brain pericytes was elevated after LPS treatment; this shows that pericytes are a source of IL-13 as well.

Additionally, compared to published results from LPS treated mouse microglia [38], production of IL1-α and TNF-alpha, a two typical proinflammatory cytokines, by brain pericytes was low. This shows that although pericytes and microglia both respond to LPS, the profile of cytokines released is different.

Recently an interesting study comparing the gene profile expression of different cell components of neurovascular unit in adult or during the development was published. The study revealed several important genes that are involved in pericyte-endothelial signaling such as transforming growth factor beta superfamily members bmp5 and nodal [39]. It would be interesting to perform such study with immune-challenged neurovascular unit as well.

Neurodegenerative processes are closely associated with neuroinflammation [40]. In Alzheimer's disease, increased production and impaired transport lead to accumulation of toxic amyloid beta peptide deposits along the vascular system in patients affected by this disease. LRP-1 at the brain endothelial cell is an important transporter for the brain-to-blood efflux of amyloid beta peptide [41] and in neurons is important in the processing of amyloid precursor protein [42,43]. It has been shown previously that human brain pericytes express LRP-1 and that the expression is increased after incubation of cells with amyloid beta peptide [44]. It is likely that pericyte LRP-1 contributes to the uptake and processing of amyloid beta peptide and amyloid precursor protein. Interestingly, accumulation of amyloid beta peptide within the pericyte bodies have been previously described for early onset familial [45,46] and for sporadic Alzheimer's disease [47]. In line with these observations, we analyzed the expression of LRP-1 in brain pericytes during brain inflammation. We demonstrated that the expression of both subunits of LRP-1 is increased in brain pericytes under inflammatory conditions.

## Conclusions

In conclusion, our results as presented here show that cultured mouse brain pericytes secreting NO, cytokines, and chemokines and responding to LPS stimulation. We also showed that pericytes in-vitro express LRP-1, an important regulator of the levels of amyloid beta peptide in the brain, and that expression is influenced by LPS. These immunoactive properties of cultured pericytes suggest mechanisms by which they can act as an integral part of the neurovascular unit during brain inflammatory processes such as brain infections and neurodegenerative processes.

## List of abbreviations

BBB: blood-brain barrier; NO: nitric oxide; LRP-1: lipoprotein receptor-related protein-1; CD11B: cluster of differentiation molecule 11B; LPS: lipopolysaccharide; GFAP: glial fibrillary acidic protein; iNOS-2: inducible NO synthase-2; MAPK: mitogen-activated protein kinase.

## Competing interests

The authors declare that they have no competing interests.

## Authors' contributions

AK designed the study, performed the bulk of the experiments and analyzed all data. AK and WB wrote the manuscript. ME performed the western blot analysis. All authors have read and approved the final version of this manuscript.
